# Rapid Evolution of Recombinant *Saccharomyces cerevisiae* for Xylose Fermentation through Formation of Extra-chromosomal Circular DNA

**DOI:** 10.1371/journal.pgen.1005010

**Published:** 2015-03-04

**Authors:** Mekonnen M. Demeke, María R. Foulquié-Moreno, Françoise Dumortier, Johan M. Thevelein

**Affiliations:** 1 Laboratory of Molecular Cell Biology, Institute of Botany and Microbiology, KULeuven, Leuven-Heverlee, Flanders, Belgium; 2 Department of Molecular Microbiology, VIB, Leuven-Heverlee, Flanders, Belgium; New York University, UNITED STATES

## Abstract

Circular DNA elements are involved in genome plasticity, particularly of tandem repeats. However, amplifications of DNA segments in *Saccharomyces cerevisiae* reported so far involve pre-existing repetitive sequences such as ribosomal DNA, Ty elements and Long Terminal Repeats (LTRs). Here, we report the generation of an eccDNA, (extrachromosomal circular DNA element) in a region without any repetitive sequences during an adaptive evolution experiment. We performed whole genome sequence comparison between an efficient D-xylose fermenting yeast strain developed by metabolic and evolutionary engineering, and its parent industrial strain. We found that the heterologous gene *XylA* that had been inserted close to an ARS sequence in the parent strain has been amplified about 9 fold in both alleles of the chromosomal locus of the evolved strain compared to its parent. Analysis of the amplification process during the adaptive evolution revealed formation of a *XylA*-carrying eccDNA, pXI2-6, followed by chromosomal integration in tandem arrays over the course of the evolutionary adaptation. Formation of the eccDNA occurred in the absence of any repetitive DNA elements, probably using a micro-homology sequence of 8 nucleotides flanking the amplified sequence. We isolated the pXI2-6 eccDNA from an intermediate strain of the evolutionary adaptation process, sequenced it completely and showed that it confers high xylose fermentation capacity when it is transferred to a new strain. In this way, we have provided clear evidence that gene amplification can occur through generation of eccDNA without the presence of flanking repetitive sequences and can serve as a rapid means of adaptation to selection pressure.

## Author Summary

Xylose is an important component of lignocellulose hydrolysates used for the production of bioethanol, but the yeast *Saccharomyces cerevisiae* is unable to utilize xylose. Insertion of a bacterial xylose isomerase gene and improvement of growth on xylose by evolutionary adaptation resulted in amplification of this gene and efficient xylose fermentation capacity. Further analysis of the final and intermediate strains from the evolutionary adaptation process revealed interesting features about the mechanisms involved in gene amplification events, which have occurred frequently in natural evolution. We now show that a circular DNA element was spontaneously created by the yeast, encompassing the xylose isomerase gene and an ARS element, present by coincidence adjacent of the inserted xylose isomerase gene. ARS elements are the sites where DNA polymerase initiates duplication of DNA. Interestingly, this has revealed for the first time in yeast that circular DNA plasmids can be created from genomic DNA in the absence of flanking repetitive sequences.

## Introduction

Microbial evolutionary experiments have received considerable attention in recent years for various reasons. First they allow in depth understanding of the fundamental process of evolution in a rapid and rigorously controlled way [1]. Second, microbial evolution raises great interest in various fields such as in medicine and industrial applications [2–4]. Using nature’s evolutionary principle of variation and selection, microbial evolution has been used for development and optimization of several production host organisms in industrial applications. The speed of fitness gain in a new environment depends on the rate of genetic changes as well as their advantage [5]. Genetic changes that occur during evolution include point mutations, gene deletions or amplifications, and often gene rearrangements involving transposable elements, which in turn might generate deletions or amplifications.

In a broader context, gene duplications and amplifications have played a crucial role in the evolution and genetic diversity of species, in particular for adaptation to restrictive environmental conditions [6,7]. Segmental duplications and amplifications are common in eukaryotes. In the yeast *Saccharomyces cerevisiae* genome, about 1 out of 5 genes have been identified as duplicates [8]. Moreover, nearly 2% of the coding sequences in *S. cerevisiae* are tandem gene arrays [9]. Tandem repetitive DNA sequences that include ribosomal DNA (rDNA) and the telomeric loci are very prone to copy number alterations as a consequence of homologous recombination (HR). Such regions play a significant role in the plasticity of the genome. Other repetitive elements like Ty elements and solo Long Terminal Repeats (LTRs) that are widely dispersed in the yeast genome are potential substrates for HR between the short repeats flanking a DNA segment. In spite of the major contribution of repetitive DNA sequences in elevated rates of genome plasticity, segmental amplifications are not restricted to regions with repetitive sequences. However, the generation of tandem gene amplifications from originally single copy sequences is not well understood. The creation of extrachromosomal circular DNA (eccDNA) has been proposed as a possible mechanism for the origin and plasticity of tandem gene repeats [10]. The formation of eccDNA has been attributed to the circularization of a DNA segment from a chromosome during HR between preexisting closely located homologous sequences such as LTRs, resulting in the excision of the DNA segment [11]. There has only been little experimental evidence for the formation of eccDNA in the absence of repeat sequences [12].

The yeast *S. cerevisiae* has a very long proven record of industrial application, due to its efficient conversion of glucose into ethanol with high productivity, and its substantial tolerance to various inhibitory compounds, including ethanol [13,14]. However, it is unable to efficiently metabolize D-xylose into ethanol. Typically, D-xylose accounts for about one-third of the sugars in lignocellulosic biomass [15,16]. Due to the recent interest in biofuel production with biomass from waste streams and bioenergy crops, engineering *S. cerevisiae* for efficient D-xylose to ethanol conversion has become an important research focus [17]. Expression of the heterologous structural genes responsible for D-xylose to ethanol conversion in *S. cerevisiae* did not lead by itself to sufficient productivity for industrial scale application [18].

Recently, using a combination of metabolic and evolutionary engineering strategies, we have developed a robust industrial yeast strain that displayed the highest D-xylose to ethanol conversion rate and yield compared to any other recombinant yeast strain reported previously [19]. Here, we report the elucidation of one of the crucial genetic changes responsible for the rapid D-xylose utilization rate in this strain. Using whole genome sequence comparison of the evolved strain with that of the parent strain, we identified a major copy number variation in the heterologous gene *XylA*, encoding *Clostridium phytofermentans* XI (xylose isomerase)), that correlated with the high enzymatic activity measured in crude cell extracts. In addition, we investigated the evolutionary process that resulted in stable integration of this gene in tandem high copy number into the genome using several intermediate strains with varying xylose fermentation rate. We confirmed the formation of self-replicating eccDNA carrying the gene *XylA* during adaptive evolution in the absence of any homologous sequence flanking the repeat DNA segment. During later stages of the adaptive evolution, the generated eccDNA had integrated into the locus of origin in the genome, generating increasing numbers of tandem repeats first in one of the chromosomes and later in the second chromosome in the diploid strain. We propose that formation of eccDNA can occur in the absence of HR and can serve as a rapid means of adjustment to selection pressure during evolutionary adaptation, especially when higher gene dosage serves as a selective advantage for proliferation or survival.

## Results

### Whole genome sequence analysis

Recently, we reported the development of an industrial D-xylose utilizing strain of *S. cerevisiae*, GS1.11–26, using a combination of metabolic engineering, genome shuffling and evolutionary adaptation [19]. Briefly, we integrated the gene *XylA* coding for Xylose Isomerase from the bacterium *Clostridium phytofermentans* together with all the known genes important for D-xylose and L-arabinose metabolism into an industrial bioethanol production strain, ER, (Ethanol Red). However, the recombinant strain named HDY.GUF5 was unable to utilize any D-xylose. We then mutagenized this strain by EMS and selected a mutant isolate M315 that was able to grow slowly on D-xylose but had poor D-xylose fermentation capacity. Genome shuffling of this mutant with its parent HDY.GUF5, followed by selection for faster D-xylose growth resulted in little improvement. Subsequently, we performed evolutionary adaptation in serial batch cultures containing D-xylose as main carbon source. A striking observation during this adaptation process was a drastic gain in D-xylose fermentation capacity at the second serial batch culture. We proposed that a crucial genetic change had happened at that step, which resulted in a rapid gain in performance. To elucidate this change, we sequenced the genome of the original parent strain HDY.GUF5 and the final evolved strain GS1.11–26 and performed a global genome sequence comparison.

### Detection and evaluation of copy-number variations (CNVs)

**Fig 1:**
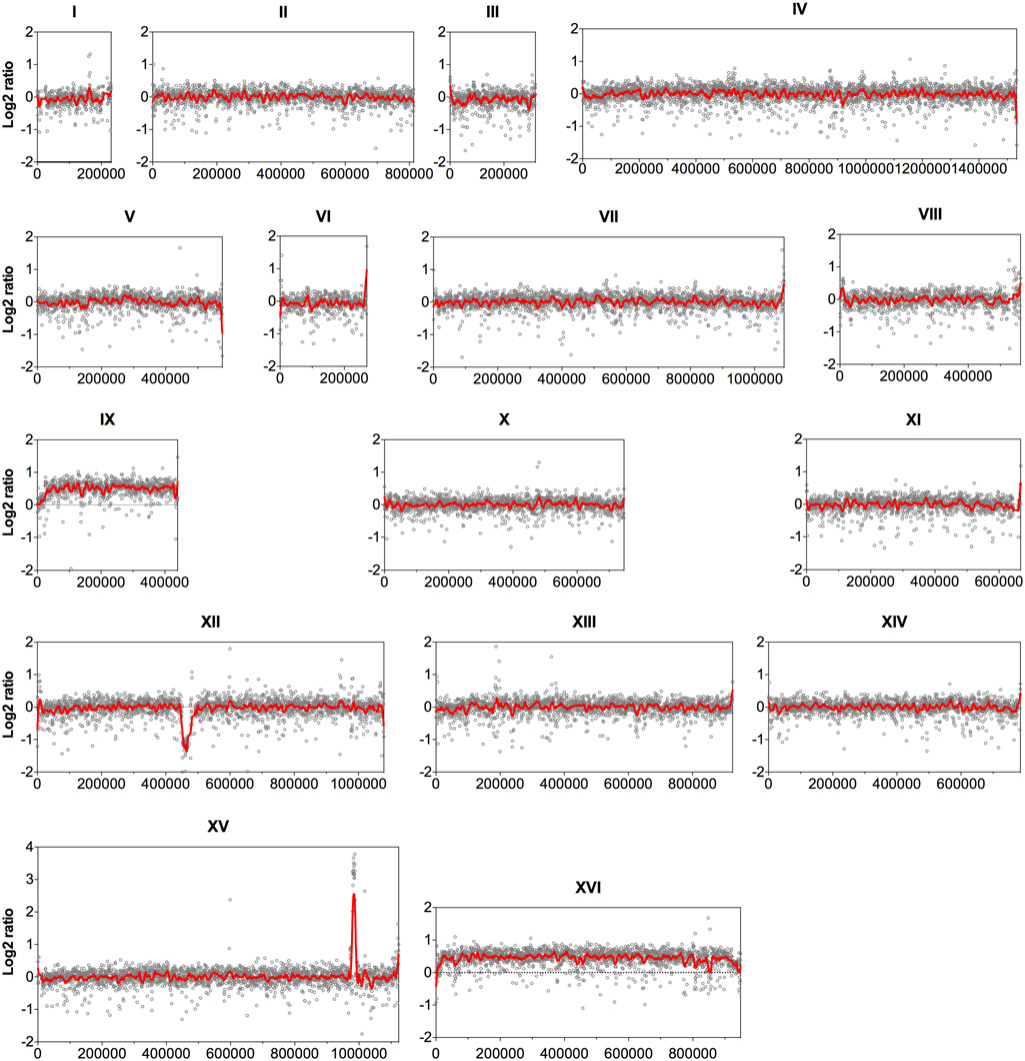
Comparison of the genome sequence coverage between the parent strain HDY.GUF5 and evolved strain GS1.11–26. Log2 ratio depicted from whole genome sequence coverage between the parental and the evolved strain is presented for each of the 16 chromosomes. Each grey circle represents the value of the log2 ratio obtained from the sequence coverage calculated for averaged sliding windows of 500 nucleotide positions. The red line indicates the smoother trend calculated by moving average values of 10,000 bp.

Since the coverage depth of the sequence reads reveals CNVs among genomes of different strains [20], the sequence coverage of all 16 nuclear chromosomes was analyzed at the nucleotide level with an average window of 500 bp. The log2 ratio of the read depth between the evolved and the parent strains was then calculated and plotted over the length of the chromosomes (Fig. 1). Accordingly, chromosome IX and chromosome XVI showed a 50% higher coverage in the evolved strain compared to the parent strain, indicating duplication of one of the two homologues for each chromosome in the evolved strain.

In chromosome XII, the coverage at the ribosomal DNA locus was reduced in the evolved strain by about 50% relative to the parent strain, indicating the loss of several copies of the ribosomal DNA (rDNA) genes. rDNA genes are present in about 150 to 200 tandem copies in the *S. cerevisiae* genome [21]. The possible effect of the reduction of the rDNA copies in our evolved strain was not investigated further. However, large deletions of multiple rDNA copies are common spontaneous phenomena in the yeast genome. Strains with up to 50% reduction in the number of rDNA copies compared to wild type laboratory strains did not show any noticeable defect in mitotic growth and meiotic reproduction [22].

**Fig 2:**
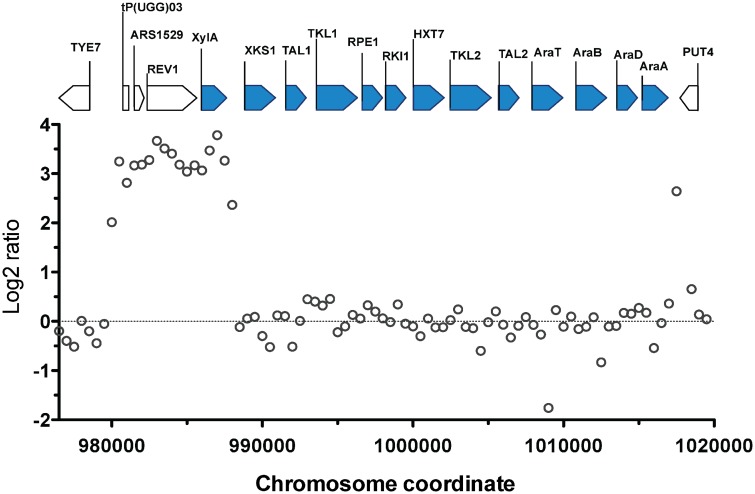
Comparison of the xylose cassette sequence coverage between the parent strain HDY.GUF5 and evolved strain GS1.11–26. Log2 ratio of sequence coverage between the evolved and parent strain in the locus of chromosome XV, where the D-xylose and arabinose gene cassette has been integrated, is plotted over the length of the chromosomal fragment. Annotations present in the locus are indicated by bars at the top of the figure. Bars shaded in blue correspond to the heterologous genes that were inserted into the chromosome, while the unshaded bars represent genes present in part of the original yeast chromosome. The coverage was computed for averaged sliding windows of 500 nucleotide positions.

The most prominent CNV occurred in a region at the right arm of chromosome XV. This was the region where the D-xylose and L-arabinose metabolism gene cassette had been integrated in the genome of the parent strain HDY.GUF5 [19]. Part of the integrated gene cassette, containing the *XylA* gene, and a sequence upstream of the integrated cassette, that includes the gene *REV1*, the tRNA gene *tP(UGG)O3* and the autonomously replicating sequence *ARS1529*, were amplified about 9 times (estimated from the log2 ratio) in the evolved strain compared to the parent strain (Fig. 2). *XylA* encodes xylose isomerase that converts D-xylose to D-xylulose, the rate-limiting step in D-xylose fermentation. We previously showed that the evolved strain GS1.11–26 displayed significantly higher (about 17 times) XI activity than the parent strain, which displayed only moderate activity [19]. The high copy number of *XylA* in the evolved strain is therefore consistent with its high XI activity, though the fold increase in the XI activity was higher than that of the copy number.

### Amplification of the *XylA-*locus as tandem repeat in GS1.11–26

**Fig 3:**
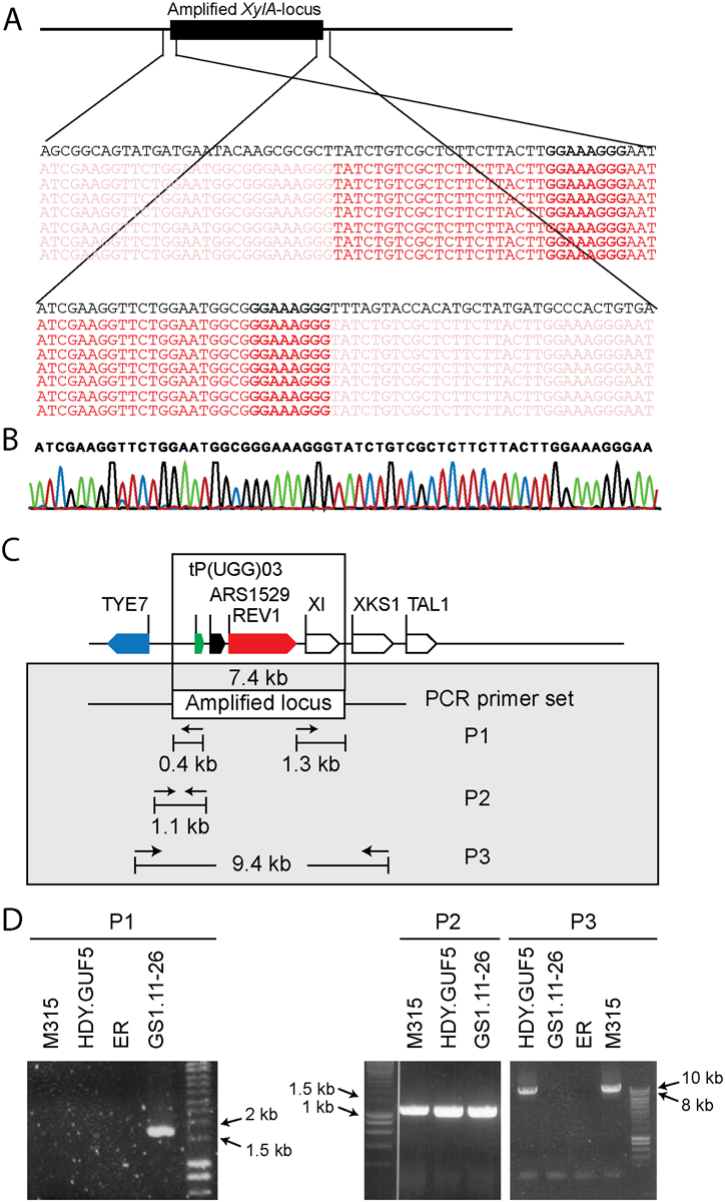
Sequence analysis at the borders of the amplified *XylA*-locus, and verification of the presence of circular or tandem repeats. (A) Illumina sequence reads mapped to the reference sequence at both ends of the amplified *XylA*-locus. Part of the reads that match to the reference sequence are shown in dark red, while sub-reads that do not match to the reference sequence are indicated in faded red color. The reference sequence is shown in black at the top of the reads. Microhomologies located on either ends of the amplified locus are shown in black rectangular boxes. (B) Chromatogram from Sanger sequencing of the amplified locus, showing continuous reads through the break point of the Illumina sequence reads alignment. The Sanger sequencing was performed using a PCR product obtained by amplification with primers that anneal outwards from both ends of the locus. The same result was obtained when the plasmid isolated from strain GS1.2–6 was sequenced. (C) Schematic representation of the amplified *XylA*-locus and PCR primer sets used. Horizontal arrows stand for annealing sites and direction of the PCR primers used to verify circular or tandem repeat formation (primer set P1), the presence of the *XylA* locus at the right position (primer set P2) and the possibility of deletion or single copy of the *XylA*-locus (P3). The size of the expected PCR product is given in kb. (D) Agarose gel electrophoresis picture of the PCR products obtained using the three sets of primers (P1, P2 and P3) with DNA samples from different strains. For primer set P3, the result obtained with a long extension time (8 min) is shown. ER (Ethanol Red) is the original industrial strain in which the xylose/arabinose gene cassette has been inserted into the genome.

We sought to understand the structural arrangement of the amplification of the *XylA*-locus. The presence of the autonomous replication sequence ARS1529 in the amplified *XylA-*locus made us consider the possibility that this region got amplified as a self-replicating eccDNA. This idea was supported by the observation of break points on either end of the amplified region when the Illumina sequence reads were mapped to the reference genome (Fig. 3A). The sequence reads at one end of the break point contained partially unmatched sequences that matched with the sequence of the opposite end. This condition implies either circular DNA or tandem repeat formation.

To validate this assumption, we performed PCR, (polymerase chain reaction) using a primer set P1, which consisted of a pair of primers inside the amplified region that project outwards in opposite direction (Fig. 3C). A PCR product of 1.7kb was expected only if a circular or tandem repeat sequence had been generated. The evolved strain GS1.11–26 gave a PCR product of the expected size, while no PCR product was obtained from the parent strain HDY.GUF5, the mutant M315 and the original industrial strain Ethanol Red that does not have the cassette (Fig. 3D). The PCR product obtained was then sequenced using conventional Sanger sequencing. The resulting contigs were shown to read through the break point that was obtained from the alignment of Illumina sequence reads at either end of the amplified region (Fig. 3B), indicating a continuous DNA sequence, which in turn points to either a circular form or a tandem repeat.

To differentiate between circular DNA and tandem repeat, we first evaluated if the chromosomal copy of the amplified region had been deleted in the evolved strain. Deletion of the locus would contradict the possibility of tandem amplification of the locus. For this purpose, two sets of primers were used (Fig. 3C). The primer set P2 contained a forward primer annealing upstream of the amplified locus and a reverse primer annealing inside the amplified locus, and detects the presence of the *XylA*-locus at the right position in the chromosome. The primer set P3, contained a forward primer upstream of the amplified locus and a reverse primer downstream of the amplified locus, and was expected to give a PCR product only upon deletion of the *XylA* locus since the locus is too large to be amplified with the PCR conditions used (2 min extension time). A band of the expected 1.1 kb size with the PCR set P2 (Fig. 3D) and a negative result with the PCR set P3 with the 2 min extension time was obtained for both the parent HDY.GUF5, the M315 mutant and the evolved strain GS1.11–26. The positive band with PCR set P2 was expected since the whole genome sequencing data indicated that at least one of the alleles was present in the locus. However, the absence of a PCR product using primer set P3 indicated that neither of the two alleles of the chromosomal *XylA*-locus was deleted. Therefore, neither tandem amplification nor eccDNA could be excluded on the basis of this PCR analysis.

**Fig 4:**
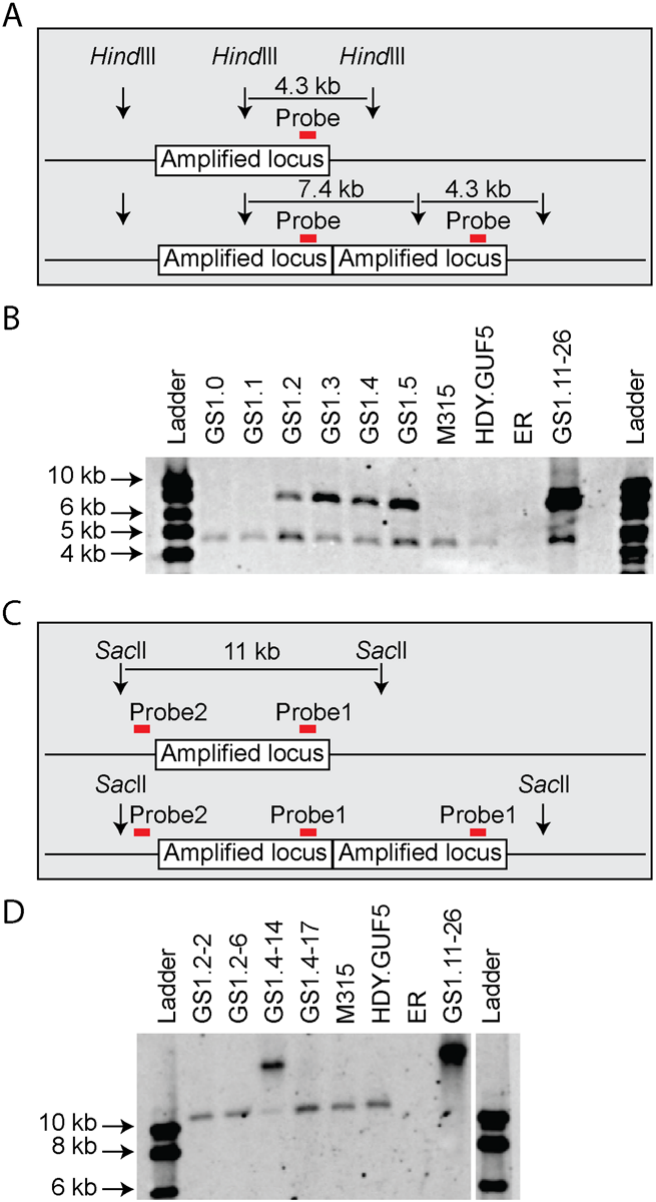
Evaluation of the amplified *XylA*-locus by Southern blot analysis. (A) and (C) show a schematic representation of the amplified *XylA*-locus in GS1.11–26. Vertical arrows represent cutting sites of the restriction enzymes; red horizontal bar indicates the locus of the unique site for probe hybridization. The amplified locus is shown once in the upper part and twice in the lower part. (B) Image of the Southern blot analysis after *Hind*III digestion. Two bands of expected size 4.3 kb and 7.4 kb were detected in GS1.11–26 and in cultures obtained from the second step of evolutionary adaptation onwards (GS1.2 to GS1.5). The parent strain HDY.GUF5, the mutant M315 and the culture from GS1.0 and GS1.1 showed only one band representing a single copy of the locus. The negative control ER, (Ethanol Red), which does not have the gene cassette, showed no band. (D) Southern blot image after digestion with *Sac*II, that cuts only outside the amplified *XylA*-locus. Two single cell isolates from GS1.2 (GS1.2–2 and GS1.2–6) and two from GS1.4 (GS1.4–14 and GS1.4–17) were evaluated. The band of about 11kb represents a single copy of the *XylA* locus, and is present in all the tested strains except the final evolved strain GS1.11–26, which showed only a high molecular weight band. GS1.4–14 showed both the 11kb and a higher molecular weight band indicating the presence of multiple copies of the locus in one allele and a single copy in the other allele. GS1.2–6 apparently lost the circular DNA during growth in non-selective condition.

We then performed PCR using the primer set P3 under conditions that allow amplification of the whole amplified *XylA*-locus (long extension time). A single copy of the *XylA*-locus in the genome was expected to produce a 9.4 kb PCR product while chromosomal duplication or amplification of the locus should not produce any PCR product since it would be too large to be amplified. The parent HDY.GUF5 and the mutant M315 strains gave rise to a PCR product with the correct size of 9.4 kb but the evolved strain GS1.11–26 did not give rise to any band after several attempts (Fig. 3D). The HDY.GUF5 positive control gave rise to the expected band in all repetitions. This indicates that only one copy of the *XylA* locus is present in each of the two alleles in the parent strain and the M315 mutant, but that the evolved strain might have multiple copies in both alleles. To confirm this assumption, Southern blot analysis was performed with genomic DNA digested with two different restriction enzymes. First, the DNA was digested with *Hind*III that cuts only once inside the amplified *XylA*-locus. A unique probe that hybridizes in the *XylA* sequence was used to visualize the band. In the presence of only a single copy of the *XylA*-locus, a single band of 4.3 kb was expected while a circular or tandem repeat sequence should give two bands of 4.3 kb and 7.4 kb (Fig. 4A). Two bands of the expected size were detected for the evolved strain, GS1.11–26. The intensity of the 7.4 kb band was estimated to be about 8 fold higher than the intensity of the lower band, which closely correlated to the amplification level deduced from the whole genome sequence analysis. In the parent strain HDY.GUF5 and the mutant M315, only the 4.3 kb band was detected indicating a single copy of each allele (Fig. 4B). No band was detected in the control strain Ethanol Red, which does not contain the gene cassette in the genome.

We then digested the genomic DNA with *Sac*II, which cuts only outside the amplified *XylA*-locus, and hybridized with two different probes annealing either inside (same as the previous probe, Fig. 4C), or outside the amplified locus (between the left *Sac*II restriction site and the amplified locus). An 11 kb band was expected if a single copy of the *XylA*-locus was present in the chromosomal locus. Accordingly, the presence of the expected 11 kb fragment in the strains HDY.GUF5 and M315 using both probes hybridizing inside (Fig. 4D) or outside the amplified *XylA*-locus confirmed the existence of a single copy of the *XylA*-locus in both alleles. On the other hand, the evolved strain showed only a higher molecular weight band, both with the inside (Fig. 4D) and outside probes confirming the presence of multiple copies of the *XylA*-locus in both chromosomal alleles. This result together with the PCR amplification using PCR set P1 (primers directed outwards on either side of the amplified locus) clearly indicates that the amplification of the locus in GS1.11–26 had occurred in the form of a tandem repeat.

### Amplification of the *XylA*-locus during evolutionary adaptation with eccDNA intermediate

**Fig 5:**
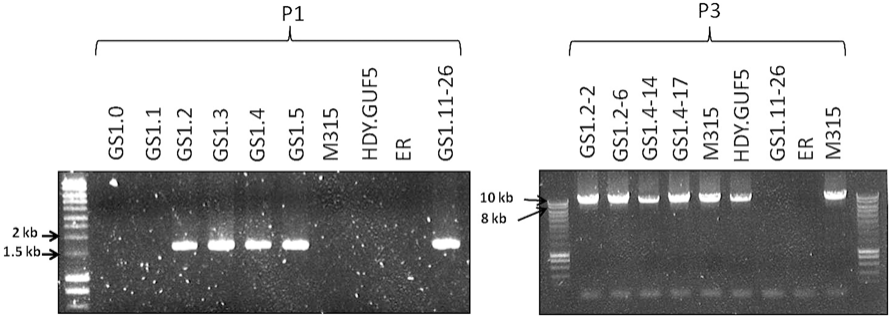
Evaluation of the *XylA-*locus in cultures and single cell clones obtained from the various stages of the evolutionary adaptation process. Agarose gel electrophoresis of PCR products obtained using primer sets P1 and P3 are shown (see Fig. 3C for the primer sets). DNA was isolated from a sample of the whole culture obtained just after the genome shuffling step (GS1.0) and after each batch of the first 5 cultures of the evolutionary adaptation process (GS1.1 to GS1.5). GS1.2–2 and GS1.2–6 are single cell isolates from the culture GS1.2, while GS1.4–14 and GS1.4–17 are single cell isolates from the culture GS1.4. The strains M315, HDY.GUF5, ER, (Ethanol Red) and GS1.11–26 have been included for comparison.

As described in our previous report [19], the evolutionary adaptation step used to obtain the strain GS1.11–26 involved a series of 11 sequential batch cultures in D-xylose medium. To verify at which stage of the evolutionary adaptation process the amplification of the *XylA-*locus had occurred, a sample from the culture before the evolutionary adaption (GS1.0), and samples at the end of the first 5 serial transfers during the evolutionary adaptation (GS1.1, GS1.2, GS1.3, GS1.4 and GS1.5) were tested by PCR for the presence of the tandem amplification or circular DNA formation using PCR primer set P1. Interestingly, a positive PCR result was obtained in all the samples derived from the second culture (GS1.2) onwards, whereas isolates from GS1.0, GS1.1, as well as the original strains used for the genome shuffling step (HDY.GUF5 and M315) did not give rise to the PCR product (Fig. 5). Southern blot analysis of the same samples after *Hind*III digestion also confirmed the presence of either circular or multiple copies of the locus in the samples obtained from GS1.2 onwards (Fig. 4B). This strongly suggests that amplification of the *XylA*-locus had occurred at the second step of the evolutionary adaptation process (GS1.2). As we anticipated, the sharp rise in the rate of D-xylose fermentation in the second culture [19], correlated with amplification of the *XylA-*locus. Although *XylA* was expressed from a strong promoter in the parent strain HDY.GUF5, the level of expression was not high enough to confer strong D-xylose fermentation capacity. Amplification of the gene likely increased the expression of XI, which in turn alleviated the rate limiting bottleneck for fermentation of D-xylose.

Remarkably, the chromosomal tandem amplification of the *XylA* locus in GS1.11–26 was not detected in two D-xylose fermenting single cell clones obtained from GS1.2 (second culture) that showed a positive PCR using the primer set P1. When the Southern blot was performed after *Sac*II digestion on these single cell isolates, called GS1.2–2 and GS1.2–6, only the 11kb band was obtained for both strains, which excludes the possibility of chromosomal amplification (Fig. 4D). This was supported by the result of the PCR amplification of the whole amplified *XylA*-locus using primer set P3, which gave only the expected 9.4 kb band (Fig. 5). Since no smaller PCR band was obtained when this primer set was used (indicating that the *XylA*-locus was not deleted), and only a single band was obtained with the Southern blot assay (Fig. 4D), both chromosomes should have a single copy of the *XylA*-locus in these two strains. Given the positive PCR result obtained using primer set P1 (Fig. 5) that indicates either circular or tandem copies of *XylA* in all the cultures obtained from GS1.2 onwards, and the absence of chromosomal tandem repeats in the culture of GS1.2 clearly indicate that a circular DNA was generated at the second stage (GS1.2) of the evolutionary adaptation process.

We also performed the Southern blot assay with *Sac*II digested DNA using genomic DNA of two other single cell isolates from GS1.4 (4^th^ culture) to test for the presence of the eccDNA. The first isolate GS1.4–14 had the highest D-xylose fermentation rate among all the isolates obtained from the culture GS1.4. Another isolate GS1.4–17 (with only moderate D-xylose fermentation capacity) was also used for comparison. Accordingly, GS1.4–14 showed both the 11kb and a higher molecular weight band (Fig. 4D). Together with the result of the PCR assay shown in Fig. 5, this result clearly indicates the presence of multiple copies of the locus in one of the alleles and a single copy in the other allele in strain GS1.4–14. The strain GS1.4–17 showed only the 11kb band in the Southern blot assay, which was also consistent with the PCR amplification of the whole *XylA-*locus using primer set P3 (Fig. 5), indicating the presence of a single copy of the *XylA*-locus in both alleles. Similar to GS1.2–6, strain GS1.4–17 gave a positive PCR result using primer set P1, indicating the presence of a circular DNA in this strain. This result also suggested a correlation between the multiple integration of the *XylA*-locus in the genome and the faster D-xylose fermentation.

Eventually, we had obtained clear indications that amplification of the *XylA*-locus had arisen through a circular intermediate in an early stage of the evolutionary adaptation process, and subsequently recombined in tandem array at the same locus in one of the chromosomes. Later, unequal crossover or other mechanisms might have led the tandem array to be copied into the second chromosome, since GS1.11–26 carried the amplified locus in both alleles.

### Stability of eccDNA in intermediate strain GS1.2–6

**Fig 6:**
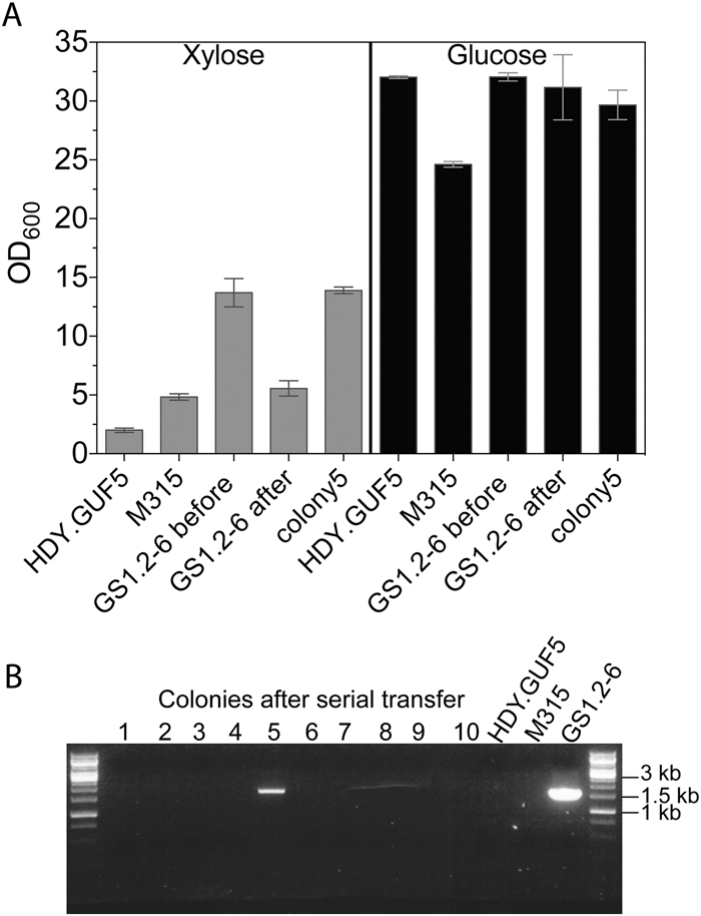
Evaluation of the stability of the *XylA*-carrying circular DNA in GS1.2–6. (A) A single cell isolate, GS1.2–6, from the GS1.2 culture, was grown in YPD medium for about 25 generations and spread for single colonies on YPD. Forty-three isolates from the serially transferred culture were tested for growth performance in YPX medium or YPD medium (as control). For comparison, the original GS1.2–6 before serial transfer in YPD, the parent HDY.GUF5 and the mutant M315 were included. The cells were inoculated into YPX medium at an initial OD_600_ of 0.5. Optical density was measured after 48 h at 30°C. From the 43 colonies tested, only colony 5 displayed efficient growth and is shown separately. The average OD value of the remaining 42 is shown as ‘GS1.2–6 after’. Error bars represent standard deviation from an average of 42 colonies for GS1.2–6 after, and at least triplicate values for the other strains. (B) Agarose gel electrophoresis picture of the PCR assay using primer set P1. The results for 10 colonies out of the 43, and for the control strains are shown. No band was detected for the remaining colonies. The bands in colony 5 and the control GS1.2–6 indicate the presence of the *XylA* carrying circular DNA.

Next, we evaluated the stability of the high xylose fermentation capacity phenotype in GS1.2–6. If the strain GS1.2–6 carried only the circular plasmid and not the genomic *XylA* amplification, the loss of the plasmid should cause loss of its high D-xylose growth capacity. To allow for loss of the plasmid, GS1.2–6 was grown in rich medium with glucose (YPD, Yeast extract peptone dextrose) for about 25 generations. The culture was spread for single colonies and 43 single cell isolates were tested for growth in liquid YPX (Yeast extract peptone D-xylose) medium. All isolates except one colony lost the ability to efficiently grow in D-xylose medium, consistent with loss of the *XylA* carrying circular DNA from GS1.2–6 (Fig. 6A). All the 43 colonies were further tested by PCR for the presence or absence of the eccDNA using primer set P1. Accordingly, the eccDNA could be detected in none of the colonies that lost the D-xylose growth capacity except in the one colony that kept the high growth efficiency in D-xylose (Fig. 6B). This indicates that the GS1.2–6 carried only the circular DNA and not the chromosomal amplification of *XylA*. The rapid D-xylose fermentation capacity by the final strain GS1.11–26 was previously shown to be stable for more than 50 generations [19]. Consequently, we concluded that the stability of the phenotype in GS1.11–26 is due to the integration of the circular DNA into the genome.

### Isolation of eccDNA from intermediate strain GS1.2–6

**Fig 7:**
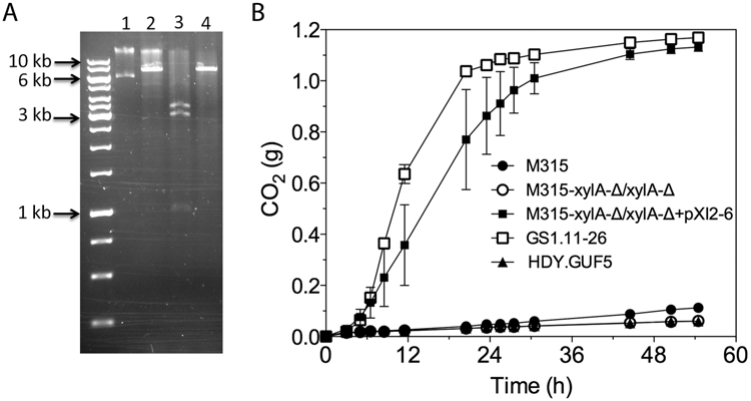
Restriction analysis and xylose fermentation conferring capacity of plasmid pXI2–6. (A) Restriction analysis of pXI2–6. Lane 1, undigested plasmid showing two bands that stand for the supercoiled (lower band) and nicked (upper band) structure; lane 2, *Xho*I digestion which has single restriction site but shows incomplete digestion; lane 3, *Sph*I and *Sac*I double digestion showing the expected 1kb, 3kb and 3.4kb band; lane 4, *Hind*III which has a single restriction site and produced the expected 7.4 kb band. (B) D-xylose fermentation performance of the mutant strain M315 expressing the plasmid pXI2–6 after deletion of the chromosomal *XylA* copies. Batch fermentation was performed in YP medium containing 4% xylose using standing fermentation bottles. The CO_2_ production was estimated from the weight loss due to conversion of xylose to ethanol and CO_2_. Error bars represent standard deviation from the mean values of triplicate experiments.

To further confirm the presence of the eccDNA, plasmid DNA isolation was performed from the strain GS1.2–6, GS1.4–14 and GS1.11–26, using a protocol modified from Singh and Weil [23]. Cells were pre-grown in 100 ml YPX medium for 24 h to enrich the pXI2–6 plasmid content in the cells. The whole 100 ml culture was used for plasmid isolation (see material and methods). As a result, a substantial amount of pXI2–6 plasmid DNA (more than 1 μg) was obtained from GS1.2–6 (Fig. 7A). On the other hand, the amount of pXI2–6 plasmid DNA obtained from GS1.11–26 and GS1.4–14 was too low to be conclusive. This is probably due to the loss of the pXI2–6 plasmid in the later steps of the evolutionary adaptation process, since there was no longer a need for the strain to maintain the plasmid when enough copies of the essential gene *XylA* had been integrated in the genome sustaining rapid D-xylose utilization. When the pXI2–6 plasmid isolated from GS1.2–6 was sequenced, a 7483 bp circular sequence was obtained, matching the size of the amplified *XylA*-locus. The complete sequence of the pXI2–6 plasmid has been provided as supplementary information (S1 Dataset). Though there were several polymorphisms compared to the corresponding sequences in the reference S288c genome, the pXI2–6 plasmid sequence was identical to that of the original parent strain obtained by Illumina sequencing. Restriction analysis using two different enzymes also confirmed the correct size of the isolated pXI2–6 plasmid (Fig. 7A).

We further tested if the isolated pXI2–6 plasmid could be transferred to a new strain and confer the strain with the ability to ferment xylose. For that purpose, strain M315 was chosen since this strain was shown to be able to ferment xylose when *XylA* was over-expressed [19]. We first deleted both chromosomal copies of *XylA* from strain M315. Deletion of *XylA* in M315 completely abolished its growth ability on xylose. Subsequently the isolated pXI2–6 plasmid was transformed into the M315 deletion mutant. We were able to select transformants based on growth on plates containing xylose as a sole carbon source. No colonies were obtained with a control plasmid devoid of the *XylA* gene. We then evaluated three independent transformants for xylose fermentation capacity. Interestingly, all three transformants carrying the isolated pXI2–6 plasmid were able to efficiently ferment xylose (Fig. 7B) indicating that the isolated pXI2–6 plasmid can be transferred to a different strain and is sufficient to confer the ability to ferment xylose efficiently. To rule out the possibility of integration of the pXI2–6 plasmid in the genome, the three transformants were then grown in YPD medium for about 20 generations and 10 independent colonies from each strain were subsequently checked for growth on xylose as sole carbon source. All of the colonies lost the pXI2–6 plasmid and the ability to grow on xylose, indicating that the *XylA* gene had not been integrated into the genome and could easily be lost in the absence of selection pressure.

### ARS1529 is a functional origin of replication in *S. cerevisiae*

**Fig 8:**
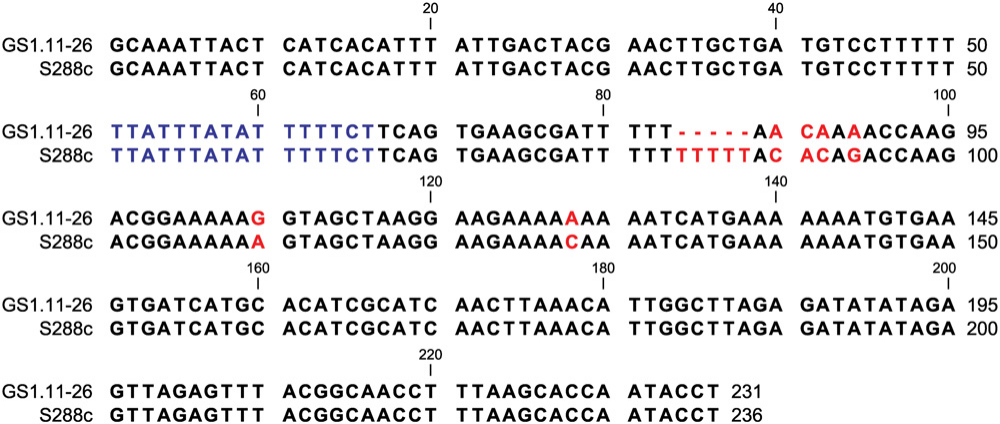
ARS1529 sequence comparison between the evolved strain GS1.11–26 and the sequence in the reference strain S288c. The nucleotide sequence in blue represents the ARS consensus sequence [24]. A gap of 5 base pairs in GS1.11–26 from bp 84 till 88 is indicated with red dashed lines. Other SNPs are also shown in red. The sequence from GS1.11–26 was confirmed by Sanger sequencing and was similar to the sequence obtained by Illumina sequencing.

ARS1529 was previously shown to be a functional replication site in yeast [24]. However, compared to the reference S288c genomic sequence, the ARS1529 sequence in the industrial parent as well as in the evolved strains contained a 5 bp deletion just 13 bp downstream of the ARS consensus sequence (ACS) and also 6 SNPs in an AT-rich region downstream of the ACS (Fig. 8). In order to validate the functionality of the modified ARS1529 version in the eccDNA intermediate, we assessed the ability of this sequence to confer self-replication. We first amplified the region containing ARS1529 together with the tRNA coding sequence and the *XylA* gene from the genomic DNA of the evolved strain GS1.11–26. The PCR product was then cloned into a yeast integrative vector containing kanMx as a selection marker. After transformation of this construct (pXI-ARS) into the mutant yeast strain M315 and selection for growth in the presence of geneticin, we obtained several transformants, with similar transformation efficiency as that of the 2μ-based plasmid, showing that the plasmid was able to propagate using ARS1529. To confirm that ARS1529 alone is sufficient to render replication capacity, we deleted all the sequences originating from the yeast genome, including the tRNA(UGG) and the XylA sequence, except the 236 bp ARS1529 sequence. Transformation of this plasmid into M315 and selection in the presence of geneticin, resulted in several transformants, which were confirmed by PCR for the presence of the plasmid. No transformants could be obtained when the same plasmid devoid of the ARS1529 sequence was transformed into M315. This resulted confirmed that the ARS1529 sequence is sufficient for plasmid replication in yeast.

## Discussion

Copy number variations are a major driving force for rapid genome evolution. The frequency of CNVs in eukaryotes is remarkably high. While many CNVs are detrimental or have no discernible effect, certain gene duplications or amplifications offer adaptive advantage under specific environmental conditions [6]. Typical examples include the copy number increase of the human salivary amylase gene AMY1, that is advantageous in a high starch diet [25] and duplication of genes coding for pepsin in Antarctic fish that allows the fish to efficiently digest at low temperature [26]. In the yeast *S. cerevisiae*, adaptation to new ecological niches has also been associated with several gene copy number variations and other chromosomal rearrangements, both in natural conditions [27,28] and in artificial evolution experiments [20,29].

In our previous report, we presented a strikingly rapid gain in D-xylose fermentation capacity observed during evolutionary adaptation of a recombinant industrial yeast strain for D-xylose utilization [19]. Here, we showed that amplification of a heterologous gene *XylA* in the form of an eccDNA, followed by reintegration in multiple tandem repeats in the genome has acted as a mechanism for rapid gain of function.

We compared the whole genome sequence of the original parent strain HDY.GUF5 that was unable to ferment D-xylose with its derivative strain GS1.11–26 that efficiently ferments D-xylose to ethanol. The strain GS1.11–26 was developed by mutagenesis, genome shuffling and evolutionary adaptation from the parent HDY.GUF5. Analysis of CNVs between the two strains revealed amplification of a chromosomal segment where the *XylA* gene had been integrated. The amplification of *XylA* correlated with elevated activity of XI. The inherently low activity of XI in the recombinant strains developed previously was a limiting factor for efficient D-xylose utilization [18]. Therefore, the high D-xylose utilization rate of the evolved strain GS1.11–26 is due, at least in part, to the high copy number of *XylA*, which resulted in high XI activity. A similar, elevated D-xylose utilization rate due to multi-copy integration of *XylA*, has been reported recently in a strain developed by expression of the *Piromyces sp*. XI and further evolutionary adaptation [30]. In that report, the original recombinant strain was constructed through expression of XI and xylulose kinase (XK) from a multi-copy plasmid. Since both genes coding for XI and XK present in the plasmid were under the same (but separate) promoter and terminator sequences (TDH3p and CYC1t), it was suggested that duplication and further copy number increase of the *XylA* gene might have been initiated though unequal crossover of homologous regions in the plasmid. However, the recombined plasmid carrying multiple copies of the *XylA* gene could not be isolated from the evolved strains due to integration of the plasmid into the genome. In our study, however, the original recombinant strain HDY.GUF5 had been constructed through direct integration of the gene cassette into the genome using an integrative vector. Moreover, we have confirmed the presence of a single copy of the *XylA* gene in both alleles of the target chromosomal locus in the strain HDY.GUF5 by whole genome sequencing and Southern blot assays. Therefore the amplification of the *XylA* locus did not start from the original plasmid that was used to construct the recombinant strain.

On the other hand, we have clearly demonstrated the occurrence of self-replicating eccDNA carrying the *XylA* gene in the course of the evolutionary adaptation process. The eccDNA carried not only the heterologous *XylA* but also a sequence upstream of the genomic locus in which it had been integrated. It included the gene *REV1* that encodes for a Deoxycytidyl transferase, an autonomous replication sequence ARS1529, and a tRNA gene *tP(UGG)O*_*3*_. The presence of these genes in the eccDNA indicates that the eccDNA was generated from the chromosomal locus. During adaptation in a serial batch culture containing D-xylose as main carbon source, a dramatic increase occurred in the rate of D-xylose fermentation just after the second transfer. The eccDNA carrying *XylA* was detected at this step in which the rapid gain of function was observed, while it was not present in the cultures of the preceding steps, indicating that the eccDNA was apparently formed in that second culture. The dramatic increase in the rate of D-xylose fermentation can now be explained by the gain of this crucial genetic change, an increased copy number of the gene *XylA* sustaining higher XI activity. We believe that formation of self-replicating eccDNA intermediates that can be maintained in the cell for several generations allows enough time (and therefore, a higher chance) for recombination to happen into the genome in multiple copies. Our results provide convincing evidence for the role of self-replicating eccDNA in the generation of tandem repeat sequences originating from a previous single copy sequence.

Selection pressure that causes a high demand for the product of a specific gene apparently results in a high chance of amplification of the locus in which the gene is located. Examples of this phenomenon have been reported previously. They include amplification of the genes *HXT6*, *SUL1* and *GAP1* in response to glucose, sulfur and nitrogen limitation, respectively [11,31–34]. In those and other studies, the explanation for the mechanism of this gene amplification has commonly been attributed to recombination between repetitive sequences flanking the gene. However, such repetitive sequences were not always present, as in the case of *SUL1* amplification under sulfur starvation [33,34]. Evidence for amplification of a DNA segment without involvement of repetitive flanking sequences was also reported in natural yeast populations and suggested to occur in the course of natural evolution [35]. The high frequency of such amplification events that do not involve repetitive sequences is not well understood.

A possible mechanism for formation of eccDNA is the involvement of transposons. Comparing the genome sequence of both the parent and evolved strains with the sequence of reference strain S288c, we noted that the reference sequence has a transposon sequence flanked by two LTRs just 3322 bp upstream of the amplified locus. However, this sequence was not present in our strain HDY.GUF5 nor in the evolved strain GS1.11–26. We also confirmed the absence of this sequence by PCR. A second solo LTR sequence inside the amplified *XylA*-locus is also present in the S288c genome but not in the strain background we used. Hence, we found no evidence for transposon mediated gene rearrangements.

Another possible mechanism involved is initial duplication by unequal sister chromatid exchange or microhomology mediated break induced repair mechanism (MMBIR). In yeast, microhomology mediated segmental duplications have been suggested to commonly occur by BIR mechanisms [36]. MMBIR occurs during DNA repair using single-stranded DNA template carrying a microhomology sequences as short as 5 to 25bp [37]. The single stranded DNA templates might be generated on several occasions: during replication, from stalled transcription complexes, or in promoter regions [36]. Analysis of the sequence of the amplified *XylA*-locus in our strain background revealed two potential sequences with microhomologies. The first 8 bp sequence homology (GGAAAGGG) is located just at the junction of the 3’ end of the amplified locus and 21 bp downstream of the 5’ end of the amplified locus (Fig. 3A). A second 7 bp homology (TATGATG) flanking the amplified locus is located 14 bp upstream and 15 bp downstream of the amplified locus. The former sequence homology is more likely to be the recombination point as it is located exactly at the break point where the DNA segment has been amplified. Since MMBIR mechanisms are known to induce duplications [36], this region might have first been duplicated and subsequently, one of the duplicated copies might be excised as a circular DNA element.

Though formation of eccDNA frequently involves terminal repeat sequences flanking a mobile DNA region [35], there was no evidence for such repeat sequences in the parent strain HDY.GUF5 nor in the evolved strain GS1.11–26 at the *XylA*-locus, that might have resulted in HR and excision. In addition, lack of evidence for deletion of the locus in any of the cultures or several single cell isolates indicated that the circular DNA was not formed by initial excision from the genome.

A common feature of previously identified gene amplifications is the presence of an ARS element in the amplified fragment. The genes *HXT6*, *SUL1*, *GAP1* and *CUP1*, of which amplification under the appropriate selective conditions has been documented [31,33,34,38], all have an ARS element adjacent to the gene. For instance, in separate evolutionary engineering experiments under sulfur limitation [33,34] all clones characterized contained an amplification of *SUL1* and none of *SUL2* (which lacks an adjacent ARS element), while both genes encode a high-affinity sulfate permease [39]. This is generally explained by the ARS element being required for eccDNA maintenance. Our work here presented clear evidence for the formation and maintenance of ARS based circular DNA during adaptive evolution.

Chromosomal integration of the eccDNA in the form of a tandem repeat occurred in our work during further evolutionary adaptation. As a consequence, the high D-xylose fermentation phenotype was completely stable for several generations without selection pressure. Multiple integration of the *XylA*-locus as tandem array in the genome seemed to further improve the D-xylose fermentation rate compared to presence of the eccDNA form. This was evident from the fact that GS1.2–6 containing only the eccDNA still fermented much slower than GS1.4–14, which has the tandem amplification in one of the alleles of the chromosomal locus. In addition, the strain GS1.11–26 that contains the amplified *XylA*-locus in both alleles, showed still faster D-xylose fermentation than GS1.4–14. This suggests that higher copy numbers of *XylA* improve D-xylose fermentation capacity. On the other hand, the possibility that other genetic changes might have arisen during the subsequent evolutionary adaptation process that contribute to the higher D-xylose fermentation rate cannot be excluded.

Gene amplification has profound effects on rapid adaptation to new ecological niches. Several of the gene amplifications documented to date are associated with preexisting duplication that involves HR. Gene amplifications arising from single copy genes without flanking homologous sequences have been proposed to be generated by MMBIR mechanisms. Given the high frequency of gene amplification events arising from initially single copy sequences, the presence of an ARS element in many of the amplified sequences reported so far, and the possibility of recombination events with only little sequence homology, self-replicating eccDNA formation followed by tandem gene amplification probably serves as a general, rapid means of adaptation to novel environments that require high expression of a specific gene.

## Materials and Methods

### Strains and growth conditions

The *S. cerevisiae* strains used in this study are listed in Table 1. Yeast cells were propagated in yeast extract peptone (YP) medium (10 g/L yeast extract, 20 g/L bacteriological peptone) supplemented with either 20 g/L D-xylose (YPX) or 20 g/L D-glucose (YPD). For solid plates, 15 g/L Bacto agar was added. For batch fermentation, synthetic complete (SC) medium (1.7 g/L Difco yeast nitrogen base without amino acids and without ammonium sulfate, 5 g/L ammonium sulfate, 740 mg/L CSM-Trp and 100 mg/L L-tryptophan) supplemented with 40 g/L D-xylose was used. For selection of strains expressing the *Kan*MX resistance marker, 200 mg/L geneticin was added to the medium. Yeast strains were maintained at -80°C in stock medium composed of YP and 26% glycerol.

**Table 1:** *S. cerevisiae* strains used in this study.

Yeast strain	Main characteristics	Source/reference
Ethanol Red	Industrial bioethanol production strain, *MAT**a**/α*	Fermentis, a division of S. I. Lesaffre, Lille, France
HDY.GUF5	Ethanol Red background, *pyk2::XylA XKS1 TAL1 TKL1 RPE1 RKI1 HXT7 AraT AraA AraB AraD TAL2 TKL2*	[19]
M315	HDY.GUF5 background; obtained by mutagenesis with 3% Ethylmethanesulfonate and selection by growth in D-xylose medium; *MATα/α*	[19]
GS1.11–26	Ethanol Red background; obtained by mutagenesis, genome shuffling and evolutionary adaptation in D-xylose medium; *MATα/α*	[19]
M315xylA-Δ/xylA-Δ	M315 strain background, both chromosomal copies of xylA deleted	This work
M315xylA-Δ/xylA-Δ+pXI2–6	M315 strain with both chromosomal copies of xylA deleted and transformed with the plasmid pXI2–6 that was isolated from GS1.2–6	This work
GS1.2–6	Single cell isolate from the second step evolutionary adaptation culture GS1.2; able to grow well in xylose	(19)

### Small-scale fermentations

Semi-anaerobic batch fermentations were performed in 100 mL SC medium containing 40 g/L D-xylose as carbon source, in cylindrical tubes with cotton plugged rubber stopper. The strains were pre-grown for 24 hours in 5ml YPD medium. For strains carrying plasmid pXI-ARS, geneticin (200 mg/L) was added to the YPD to maintain the plasmid in the strains. The pre-culture was transferred to 50 ml YPD (+ geneticin) and grown to early stationary phase. Cells were harvested and fermentation was started by inoculating the pellet to an initial OD_600_ value of 5 into 100 ml SC + 4% xylose. The fermentation cultures were continuously stirred magnetically at 120 rpm and incubated at 35°C. The profile of the fermentation was followed based on the rate of CO_2_ production at different time intervals during the fermentation period. The CO_2_ production rate was estimated by measuring the weight loss of the fermentation tubes due to CO_2_ release.

### Molecular biology methods

Yeast cells were transformed with the LiAc/SS-DNA/PEG method [40] or by electroporation [41]. Genomic DNA from yeast for PCR amplification was extracted using the PCI [phenol/chloroform/isoamyl-alcohol (25:24:1)] method [42]. PCR was performed with Phusion DNA polymerase (New England Biolabs) for construction of the vectors and sequencing purposes, and ExTaq polymerase (Takara) for diagnostic purposes. Sanger sequencing was performed by the Genetic Service Facility of the VIB, Belgium.

### Genomic DNA isolation and whole genome sequencing

The genomic DNA was extracted using a standard protocol [43]. About 6 μg high quality DNA samples were sent for sequencing to BGI (Hong Kong). The sequencing was conducted by the facility using high-throughput Illumina sequencing technology. A paired end sequence library of 500 bp was constructed and sequence reads of 90 bp were generated. The sequencing reads provided from BGI were aligned to the reference S288c genome sequence using CLC Genomics Workbench5. Out of the 6 million reads with average length of 89.2 bp, 99% matched to the reference sequence when a 93% sequence similarity parameter was used. Additionally, 98% of the reference sequence has been covered with an average coverage depth of 44. The coverage depth per nucleotide position was extracted from the alignment and plotted using GraphPad prism software.

### Southern blot analysis

Genomic DNA digested with the appropriate restriction enzyme was run on 0.8% agarose gel overnight at 50 V. A specific probe was prepared by PCR amplification from genomic DNA. The probe was labeled using Amersham Gene Images AlkPhos Direct labeling and detection system (GE Healthcare). The labeled probe was immediately used to hybridize the DNA that was blotted on a nylon membrane. Chemifluorescent signal was generated and detected using CDP-Star as a substrate in conjugation with LAS-4000 luminescent image analyzer.

### Isolation of eccDNA from intermediate strain GS1.2–6

Plasmid DNA isolation was performed from the strain GS1.2–6, GS1.4–14 and GS1.11–26, using a modified protocol from [23]. Cells were pre-grown in 100 ml YPX medium for 24 h to enrich for the plasmid. The pellet from the 100 ml culture was divided into two and each pellet was resuspended in 5 ml buffer P1 from the QIAGEN plasmid purification kit. Freshly prepared lyticase solution (1.2 M sorbitol, 0.1 M Na_3_PO_4_ buffer pH 7.4 and 1 mg/ml lyticase) was added to the mixture and incubated for 45 min at 37°C. Once the cell lysate was obtained at this step, the protocol from the QIAGEN plasmid Maxi kit was followed.

### Data access

All sequence data have been deposited in the Sequence Read Archive (SRA) at the National Center for Biotechnology Information (NCBI) and can be accessed with references SRX651886 for Samplen56 (HDY-GUF5) and SRX647780 for Samplen57 (GS1.11–26).

## Supporting Information

S1 Dataset Complete DNA sequence of the pXI2–6 plasmid. (PDF)
