# Supplementary material for: Rapid Evolution of Recombinant Saccharomyces cerevisiae for Xylose Fermentation through Formation of Extra-chromosomal Circular DNA
Source: PLoS Genet. 2015 Mar 4;11(3):e1005010. doi: 10.1371/journal.pgen.1005010 (PMC4352087; doi:10.1371/journal.pgen.1005010)
Supplement: S1 Dataset — (PDF) [file pgen.1005010.s001.pdf]

```
LOCUS       pXI2-6      7483 bp    DNA     circular
FEATURES             Location/Qualifiers
     tRNA            783..887
     Gene            1924..4881
                     /label=REV1
     Promoter        5329..5722
                     /label=pHXT7
     Gene            5723..7039
                     /label=optXI
     Terminator      7052..7312
                     /label=tCYC
  ORIGIN
        1 TATCTGTCGC TCTTCTTACT TGGAAAGGGA ATCATATTGC CCGACAGGGT GCTGACGGGA
       61 GCCGCATTAA ATAAGCATAC GAAAATCATC AAAGTTATCG GCATTTAATT TTTTAGAGAC
      121 TTCTTTATAT CCCATAGTTA GATTATTCAA TCAAAAGGTT GTAAATGCCT ATTAAGTTGC
      181 CTCTTGTCAT GCATGGTCCC ATATTCCCTG AGGCATTCCT GCCAAGAATA ATGGATTTCT
      241 TTCATGCATA ATTATTGAGT ATTTGAATCA TCTACATCGC ACCCAAAGAA ATTTGATATC
      301 GTATAATTCG GCATATTATT ATATACTGTG TAAAAAATGA CATGGGAATT AAGAAACAAT
      361 CATCAGATTT AGTGGATGCC GCAATACAAG GACTGATAAT GTAAAGAAAA AAAAGAGGAC
      421 GCGACACAAA ATGCAAGGAA AAATAATAAA TGTTACGTAG AAATATCGGT TTCCTTTCCT
      481 TGATTTCTAT ATCTTAGTAA TGAACTTCAA GAATATGCTT ATTTTACCCA ATTATAGCTT
      541 TAATCATCAA TAGGATCTTA AAAATTATTC TAATATCCAC CGAATTTTCG AAAAGTATAC
      601 TAATTTAAAC ATTTGGTGTA AGCGTTTAGG TTGCTAACGT AGCAGCAAAC GTTGGTATCA
      661 ATCACCACTT ATTGCGCTAC ATTTCAACAA TGTTCTCTTC AACTATTCGT TTTTGGGACT
      721 GAGGAAAATG AATATAAAAT TATAATACTT CATCTTGAAC TTTGAGAAAT CACAAATTGT
      781 ATGGGCGTGT GGTCTAGTGG TATGATTCTC GCTTTGGGCG ATCCCCCTGA TCAAACAGGA
      841 GGAACAAAGC ATGCGAGAGG CCCTGGGTTC AATTCCCAGC TCGCCCCTTT TTTTTGCAAC
      901 TTCGTTTTAG ACAGTTACTT AATTTTGTTT CTTAGGCACA ACAATATTTA TAAAAGAAGA
      961 ATGTAAGTAT GACGACGAAA ACGAAAAGAC TAATAGTTTC ATATAATACT AAAAGATTTC
     1021 AATTTTCGTT TTATATCCTT CCTATTCATA AATTTTATTT CGCTATGCCA ATAATGAGTG
     1081 GATGTATAGT TGATATCTGT TTCGATAATC TTAAAGTAAA TAGTAAAATC TTACTGACAT
     1141 GAATAATCCG TCCAAAACTA GATTACTGTA TACAAGCATA TTTAAAAAAA TAATGAAACT
     1201 ATGATCAAAA AACTGTGCTA CTACAGCGGT GTTGTTATCC GATACAACCG GATATTTTTC
     1261 TTTTAATGAG TCTAAACCGT GATAGCTTCA GGTTAATACA ATCAAAAAAA GCTCAAATAT
     1321 TCTTTTAATG TCGTGTTCAC AGATTCCAAT TGAATACAAC TAGGTAGTTC ATTGTATGAA
     1381 GCCTTTGCTA CGATTTTTCA CTATTGTCTG CCTTCACCTT AATGCAGACA TCCACATATT
     1441 TTAATCACTT TAAAATAAAA AGGAAGATAT ATTAGAAGTT ATAATCCAAT CTGTAAGCCA
     1501 GATTAAAATT AACGAACTCT TCTTTCATTT GAATTGAATG CTTTGAGCTG GGGTAGGTTA
     1561 TCGCAAATTA CTCATCACAT TTATTGACTA CGAACTTGCT GATGTCCTTT TTTTATTTAT
     1621 ATTTTTCTTC AGTGAAGCGA TTTTTAACAA AACCAAGACG GAAAAAGGTA GCTAAGGAAG
     1681 AAAAAAAAAT CATGAAAAAA ATGTGAAGTG ATCATGCACA TCGCATCAAC TTAAACATTG
     1741 GCTTAGAGAT ATATAGAGTT AGAGTTTACG GCAACCTTTA AGCACCAATA CCTTTTGGCA
     1801 TAGCCTAAAG ACCTGGTTCT TAATTTTAAA CAAATTTAAC TAAAGATTTC CCTATCAAAG
     1861 AAGTAACGAG TTGATAGATT TTCTCAAAAT AAATCGATAC TGCATTTCTA GGCATATCCA
     1921 GCGATGGGTG AACATGGTGG TCTTGTAGAT TTATTAGACA GCGATTTGGA ATACTCTATA
     1981 AATAGGGAAA CTCCTGATAA AAACAATTGC CTTAGCCAGC AAAGTGTCAA TGATTCACAT
     2041 TTAACAGCAA AGACCGACGG CTTGAATGCA AGGTCTTTTC TATCTACGTT AAGCGATGCT
     2101 TCCTTAATTG AATATGTCAA CCAACTCTCC CAAACTAATA AGAATAATTC TAATCCAACT
     2161 GCAGGTACTT TAAGATTTAC TACTGAAAAT ATTAGCTGTG ATGAATTACA TGCTGATCTT
     2221 GGCGGTAGCG AAGATTCACC CATAGCTCGT AGCGTTATCG AGATCCAGGA AAGTGATAGC
     2281 AATGGTGATG ATGTTAAAAA AAATACTGTG TATACTAGGG AGGCTTATTT CCACGAGAAG
     2341 GCGCAAGGAC AAACCCTGCA AGATCAAATA TTAAAAGATC AATATAAAGA TCAAATTTCT
     2401 AGTCAAAGCA GTAAAATATT CAAAAATTGC GTCATCTATA TAAATGGCTA CACCAAACCT
     2461 GGAAGACTGC AATTACACGA GATGATAGTT TTACATGGCG GAAAATTTTT ACACTATTTG
     2521 TCTTCAAAGA AAACGGTTAC TCACATAGTG GCTTCCAATT TACCATTAAA GAAAAGGATT
     2581 GAATTTGCAA ATTACAAAGT AGTCAGTCCG GATTGGATAG TTGATAGCAT CAAAGAAGCA
     2641 AGATTATTGC CCTGGCAAAA TTACTCGTTA ACATCTAAAC TTGATGAACA GCAAAAAAAA
     2701 CTAGATAATT GTAAAACCGT AAATTCAATT CCATTGCCCT CAGAGACTAG CCTGCACAAA
     2761 AGATCTAAAT GTGTAGGGTC GGCATTGTTG CCAGTAGAAC AGCAGTCACC AGTAAATCTC
     2821 AATAATTTGG AGGCGAAAAG GATAGTCGCT TGTGATGATC CTGATTTCCT TACTTCCTAT
     2881 TTTGCTCATT CGAGATTACA TCATCTCTCC GCATGGAAGG CCAATCTGAA AGATAAATTT
     2941 CTGAATGAAA ACATCAACAA GTACACCAAA ATTACGGATA AGGATACCTA CATTATCTTT
     3001 CATATCGATT TTGACTGTTT TTTTGCAACT GTTGCGTATC TATGTAGAAG TTCTAGTTTC
```

```
3061 TCAGCATGTG ATTTTAAACG GGATCCTATA GTGGTATGCC ATGGTACTAA AAACTCCGAT
3121 ATAGCTAGTT GCAATTATGT AGCAAGGTCA TATGGGATTA AAAATGGAAT GTGGGTGTCT
3181 CAAGCTGAAA AGATGTTGCC AAATGGGATC AAGCTAATAT CATTACCATA TACCTTTGAG
3241 CAATTTCAAT TAAAATCGGA AGCCTTTTAC AGTACTCTCA AAAGATTGAA CATATTCAAT
3301 TTGATTTTAC CTATATCTAT TGATGAAGCT GTTTGTGTGA GGATAATCCC TGATAATATT
3361 CATAACACTA ATACCTTAAA TGCAAAACTG TGCGAAGAAA TACGCCAAGA AATTTTTCAA
3421 GGAACGAATG GTTGCACAGT GAGCATTGGA TGTTCCGATT CTCTTGTGTT AGCAAGACTA
3481 GCTCTCAAAA TGGCGAAACC AAATGGTTAC AATATCACAT TTAAGAGTAA CCTATCTGAA
3541 GAATTCTGGT CTAGTTTCAA ACTAGATGAT CTGCCTGGAG TTGGGCATTC TACTCTGTCA
3601 AGGTTAGAAT CAACGTTTGA TAGCCCACAT TCTTTAAATG ACTTGAGAAA AAGATATACT
3661 TTAGATGCTT TGAAGGCGAG CGTCGGCTCT AAGTTAGGTA TGAAGATTCA TCTCGCGTTA
3721 CAAGGCCAAG ATGATGAAGA AAGCTTGAAA ATACTGTACG ATCCCAAAGA AGTCTTACAA
3781 AGAAAATCAC TATCAATTGA TATCAATTGG GGAATCAGAT TTAAAAATAT CACTCAGGTA
3841 GACTTGTTCA TAGAAAGAGG TTGTCAGTAT CTTTTAGAAA AATTGAATGA AATCAACAAA
3901 ACAACGTCAC AAATCACATT AAAACTGATG AGAAGATGTA AAGATGCCCC AATTGAACCC
3961 CCAAAATATA TGGGGATGGG AAGGTGCGAC TCATTCAGTC GGAGCAGCAG ATTAGGTATT
4021 CCAACAAACG AATTCGGAAT TATTGCTACC GAAATGAAAA GTTTGTATCG AACTTTGGGC
4081 TGCCCTCCAA TGGAATTAAG AGGTCTCGCT CTACAATTCA ACAAATTGGT TGATGTGGGT
4141 CCGGATAACA ATCAGCTGAA ACTGAGGTTA CCGTTTAAAA CAATAGTGAC GAACAGAGCT
4201 TTCGAAGCCT TACCGGAAGA TGTAAAAAAT GACATTAACA ACGAGTTCGA GAAAAGAAAT
4261 TATATGAGAA AAGAATCCGG GTTGACTTCA AACTCATTGA GCTCTAAAAA AAAAGGATTT
4321 GCCATTTCCA GATTAGAAGT AAATGATTTG CCCAGTACTA TGGAAGAACA GTTTATGAAT
4381 GAACTGCCAA CCCAAGTTCG AGCAGAAGTA AGACACGATT TGAGAATTCA GAAAAAAATC
4441 CAACAGACAA AGTTAGGAAA CCTACAAGAA AAGATAAAAA GGAGAGAAGA GAGCCTACAG
4501 AACGAGAAAA ATCATTTCAT GGGCCAAAAT AGTATATTCC AGCCGATCAA ATTCCAAAAT
4561 CTGACACGGT TCAAAAAAAT TTGTCAATTA GTGAAACAAT GGGTTGCCGA AACTTTAGGT
4621 GATGGAGGGC CGCATGAAAA AGATGTTAAA TTATTCGTGA AATATTTGAT TAAACTTTGC
4681 GATTCTAATA GAGTCCATTT AGTTCTTCAT TTATCAAACC TAATATCAAG GGAATTAAAT
4741 CTCTGCACCT TTTTAAATCA GGATCATTCA GGCTTCCAAA CGTGGGAAAG AATTTTACTC
4801 AATGATATAA TTCCACTTTT AAACAGAAAT AAACATACTT ACCAGACTGT GCGTAAACTT
4861 GACATGGACT TTGAAGTTTG AATTAAAAAA AAAAACAACA TTTCAGCATA CAGTAAACAC
4921 GCAGTTTGCG AACATTACCT GTAATATCTG CATGACTATT ACCAAAGAAA AAAAAAATAA
4981 CTGACACAAT GGACAATTAA ATAAAAATAA GTAAAAAAAA TAAGGACTTT AATTTTTACT
5041 AGAATTCTTG ACCAACAGTA GAAATGCGTA AGGTATTGGA ATGGCCGATT CCTCCTTTGA
5101 ATCCTTGAAT GGAAACAACA GTATCACTGT TGTCCACCAT TCCGAAAGAC CTCGCCATTT
5161 CAACACCAAA CTTTAGGCGC CTATGAACAT CCTCACCCCA GTCGTCTAGG CGTTTCGGTT
5221 CATACAGAAA TGGGAAAACA CCTCTATACA AATGCGCAAT TTTTGCCGTT CTTGCGTGTC
5281 TTGTTACTAA TATGATAGGG CAGCTTGGTC TATACGTATA CCCTTAGGGA GCTCGTAGGA
5341 ACAATTTCGG GCCCCTGCGT GTTCTTCTGA GGTTCATCTT TTACATTTGC TTCTGCTGGA
5401 TAATTTTCAG AGGCAACAAG GAAAAATTAG ATGGCAAAAA GTCGTCTTTC AAGGAAAAAT
5461 CCCCACCATC TTTCGAGATC CCCTGTAACT TATTGGCAAC TGAAAGAATG AAAAGGAGGA
5521 AAATACAAAA TATACTAGAA CTGAAAAAAA AAAAGTATAA ATAGAGACGA TATATGCCAA
5581 TACTTCACAA TGTTCGAATC TATTCTTCAT TTGCAGCTAT TGTAAAATAA TAAAACATCA
5641 AGAACAAACA AGCTCAACTT GTCTTTTCTA AGAACAAAGA ATAAACACAA AAACAAAAAG
5701 TTTTTTTAAT TTTAATCAAA AAATGAAGAA CTACTTCCCA AACGTTCCAG AAGTTAAGTA
5761 CGAAGGTCCA AACTCTACCA ACCCATTCGC TTTCAAGTAC TACGACGCTA ACAAGGTTGT
5821 TGCTGGTAAG ACCATGAAGG AACACTGTAG ATTCGCTTTG TCTTGGTGGC ACACCTTGTG
5881 TGCTGGTGGT GCTGACCCAT TCGGTGTTAC CACCATGGAC AGAACCTACG GTAACATTAC
5941 CGACCCAATG GAATTGGCTA AGGCTAAGGT TGACGCTGGT TTCGAATTGA TGACCAAGTT
6001 GGGTATTGAA TTCTTCTGTT TCCACGACGC TGACATTGCT CCAGAAGGTG ACACCTTCGA
6061 AGAATCTAAG AAGAACTTGT TCGAAATTGT TGACTACATT AAGGAAAAGA TGGACCAAAC
6121 CGGTATTAAG TTGTTGTGGG GTACCGCTAA CAACTTCTCT CACCCAAGAT TCATGCACGG
6181 TGCTTCTACC TCTTGTAACG CTGACGTTTT CGCTTACGCT GCTGCTAAGA TTAAGAACGC
6241 TTTGGACGCT ACCATTAAGT TGGGTGGTAA GGGTTACGTT TTCTGGGGTG GTAGAGAAGG
6301 TTACGAAACC TTGTTGAACA CCGACTTGGG TTTGGAATTG GACAACATGG CTAGATTGAT
6361 GAAGATGGCT GTTGAATACG GTAGAGCTAA CGGTTTCGAC GGTGACTTCT ACATTGAACC
6421 AAAGCCAAAG GAACCAACCA AGCACCAATA CGACTTCGAC ACCGCTACCG TTTTGGCTTT
6481 CTTGAGAAAG TACGGTTTGG AAAAGGACTT CAAGATGAAC ATTGAAGCTA ACCACGCTAC
6541 CTTGGCTGGT CACACCTTCG AACACGAATT GGCTATGGCT AGAGTTAACG GTGCTTTCGG
6601 TTCTGTTGAC GCTAACCAAG GTGACCCAAA CTTGGGTTGG GACACCGACC AATTCCCAAC
6661 CGACGTTCAC TCTGCTACCT TGGCTATGTT GGAAGTTTTG AAGGCTGGTG GTTTCACCAA
6721 CGGTGGTTTG AACTTCGACG CTAAGGTTAG AAGAGGTTCT TTCGAATTCG ACGACATTGC
6781 TTACGGTTAC ATTGCTGGTA TGGACACCTT CGCTTTGGGT TTGATTAAGG CTGCTGAAAT
```

```
6841 TATTGACGAC GGTAGAATTG CTAAGTTCGT TGACGACAGA TACGCTTCTT ACAAGACCGG
6901 TATTGGTAAG GCTATTGTTG ACGGTACCAC CTCTTTGGAA GAATTGGAAC AATACGTTTT
6961 GACCCACTCT GAACCAGTTA TGCAATCTGG TAGACAAGAA GTTTTGGAAA CCATTGTTAA
7021 CAACATTTTG TTCAGATAAC TCGAGTCATG TAATTAGTTA TGTCACGCTT ACATTCACGC
7081 CCTCCCCCCA CATCCGCTCT AACCGAAAAG GAAGGAGTTA GACAACCTGA AGTCTAGGTC
7141 CCTATTTATT TTTTTATAGT TATGTTAGTA TTAAGAACGT TATTTATATT TCAAATTTTT
7201 CTTTTTTTTC TGTACAGACG CGTGTACGCA TGTAACATTA TACTGAAAAC CTTGCTTGAG
7261 AAGGTTTTGG GACGCTCGAA GGCTTTAATT TGCGGCCGGT ACCCAATTCG CCCCTTAGGC
7321 GGCCGCTGTT TGCAAAAAGA ACAAAACTGA AAAAACCCAG ACACGCTCGA CTTCCTGTCT
7381 TCCTATTGAT TGCAGCTTCC AATTTCGTCA CACAACAAGG TCCTAGCGAC GGCTCACAGG
7441 TTTTGTAACA AGCAATCGAA GGTTCTGGAA TGGCGGGAAA GGG
//
```
